# Involvement of β-chemokines in the development of inflammatory demyelination

**DOI:** 10.1186/1742-2094-2-7

**Published:** 2005-02-24

**Authors:** Ileana Banisor, Thomas P Leist, Bernadette Kalman

**Affiliations:** 1 SLRHC, Columbia University, New York, NY, USA; 2 Thomas Jefferson University, Philadephia, PA, USA

## Abstract

The importance of β-chemokines (or CC chemokine ligands – CCL) in the development of inflammatory lesions in the central nervous system of patients with multiple sclerosis and rodents with experimental allergic encephalomyelitis is strongly supported by descriptive studies and experimental models. Our recent genetic scans in families identified haplotypes in the genes of CCL2, CCL3 and CCL11-CCL8-CCL13 which showed association with multiple sclerosis. Complementing the genetic associations, we also detected a distinct regional expression regulation for CCL2, CCL7 and CCL8 in correlation with chronic inflammation in multiple sclerosis brains. These observations are in consensus with previous studies, and add new data to support the involvement of CCL2, CCL7, CCL8 and CCL3 in the development of inflammatory demyelination. Along with our own data, here we review the literature implicating CCLs and their receptors (CCRs) in multiple sclerosis and experimental allergic encephalomyelitis. The survey reflects that the field is in a rapid expansion, and highlights some of the pathways which might be suitable to pharmaceutical interventions.

## Introduction

Multiple sclerosis (MS) is a disabling disease of the central nervous system (CNS) with features of autoimmunity and neurodegeneration. Although the identity of primary antigenic determinant(s) is uncertain, an interaction between β-chemokine ligands and their receptors plays a central role in the recruitment and retention of inflammatory cells in the CNS. Thus, both the disease relevant chemokine ligands and their receptors represent potential therapeutic targets in MS.

Chemokines are a group of small, structurally related chemoattractant molecules that regulate cell trafficking through interactions with a set of receptors [1]. Evidence suggests that the migration of autoreactive immune cells via the blood-brain barrier (BBB) is an early and critical process during the development of inflammatory CNS lesions of experimental allergic encephalomyelitis (EAE) and MS, and that this transmigration is regulated by chemokines produced at the blood-brain barrier (BBB) and in the CNS. Subcellular signals induced by the binding of chemokines to their G-protein-coupled receptors leads to an increased avidity of integrins on leukocytes to their corresponding receptors on endothelial cells, followed by a facilitated migration of leukocytes towards the chemokine gradient in the CNS [2,3].

In addition to chemotaxis, chemokines are also involved in the regulation of T cell differentiation, apoptosis, cell cycle, angiogenesis and metastatic processes. Further, chemokines can control the generation of soluble inflammatory products such as free radicals, nitric oxide, cytokines and matrix metalloproteases [1,4]. Considering the predominantly T helper type 1 (TH1) mediated process of inflammatory demyelination and the TH2 driven suppression of inflammation, the differential effects of various chemokines on TH1 or TH2 polarization may have particular significance. The currently known, approximately 50 chemokine genes in humans are divided into four subfamilies on the basis of characteristic patterns of cysteine residues close to the N-terminal end of the products. The CC chemokine ligand family (CCL) (also known as β-chemokines or Small Cytokine Group A – SCYA in mice) is characterized by two adjacent cysteines, while the CXC (SCYB) and CX_3_C (SCYD or fractalkine) chemokine families have one or three intervening amino acids, respectively, between the two cysteines. In the XC family (SCYC or lymphotactin), only one cysteine is present [1]. All four classes of chemokines play important roles in the immune inflammatory network, but because of the complexity of interactions, here we only discuss the CC chemokine family. In humans, there are 27 CC chemokines, most of which including CCL2, CCL7, CCL11, CCL8, CCL13, CCL1, CCL5, CCL16, CCL14, CCL15, CCL23, CCL18, CCL3 and CCL4, respectively, are encoded as a cluster within chromosome 17q11. The genes for CCL27, CCL19 and CCL21 are located within chromosome 9p13, while CCL17 and CCL22 are encoded at 16q13. The remaining CCL genes can be found on chromosome 2 and 7 [1].

A functional classification was also proposed to distinguish between lymphoid and inflammatory chemokines [1,5]. Lymphoid or homeostatic chemokines (e.g. CCL21, CCL25, CXCL13) are constitutively expressed and control physiologic trafficking of cells of the adoptive immune system during hematopoiesis and immunosurveillance. Inflammatory or induced chemokines (e.g. CCL2, CCL3, CCL5, CCL7, CCL8, etc...) are transcriptionally regulated during inflammation and mediate the recruitment of inflammatory cells to target tissues.

The effects of chemokines are mediated by G-protein coupled receptors with seven-transmembrane-domains. Chemokine receptors tend to bind multiple chemokine ligands and vice versa. However, the biologically most efficient interaction often occurs between a receptor and its primary ligand (e.g. CCL2 – CCR2). The receptor binding involves high affinity interactions and signal transduction initiated by the dissociation of G-protein complex into Gα and Gβγ subunits. Gα induces the activation of the phosphoinositidine 3-kinase pathway, while the Gβγ subunits activate phospholipase C and induce Ca^2+ ^influx and protein kinase C activation. The involvement of MAP kinases as well as JAK/STAT signaling also has been shown [6]. As of today, 10 CC chemokine receptors (CCRs), 6 CXCRs, one CX_3_CR1 and one XCR1 are known [1,6].

This review focuses on the immunomodulatory effects of the β-chemokine or CCL family in EAE and MS. CC chemokines predominantly are involved in the recruitment of monocytes / macrophages and dendritic cells (monocyte chemoattractant proteins -MCP-1 [CCL2], MCP-2 [CCL8], MCP-3 [CCL7], MCP-4 [CCL13] and macrophage inflammatory proteins – MIP-1α [CCL3] and MIP-1β [CCL4]), and to lesser degrees, T lymphocytes and NK cells (MCP and MIP chemokines, regulated upon activation normal T cell expressed and secreted cytokine [RANTES]) or occasionally other cell types (e.g. eosinophil chemotactic protein – eotactin [CCL11]) into inflammatory lesions of MS.

## Genetic evidence for the involvement of β-chemokines in multiple sclerosis

A meta-analysis of raw genotype data from three genome scans in MS families revealed the highest nonparametric linkage (NPL) score = 2.58 at 17q11 [7]. Among several candidate genes (e.g. NOS2A, OMG, NF1), a cluster of evolutionarily closely related β-chemokine genes [CCL2, CCL7, CCL11, CCL8, CCL13, CCL1, CCL5, CCL16, CCL14, CCL15, CCL23, CCL18, CCL3 and CCL4, respectively] is encoded within a 1.85 Mb segment of 17q11.2-q12. Our recent linkage disequilibrium mapping confined the susceptibility regions to 3–30 kb haplotypes defined by single nucleotide polymorphisms (SNP) within the genes of CCL2, CCL11-CCL8, CCL8-CCL13, CCL13 and CCL3 [8]. A second study is under way to confirm and further refine the MS relevant haplotypes, and then, to identify the specific disease causing nucleotide variants in an independent set of families.

Within the orthologous mouse chromosome 11, two quantitative trait loci (QTL), *eae6 *and *eae7 *were identified. While these loci control the severity and duration of EAE, *eae7 *is also a susceptibility locus for the monophasic remitting / non-relapsing subtype of the disease [9]. Sequence polymorphisms within the genes of *Scya1 *(TCA-3 or CCL1), *Scya2 *(MCP-1 or CCL2) and *Scya12 *(MCP-5 or CCL12) in *eae7 *showed striking segregations among mouse strains resistant or susceptible to EAE.

Using an advanced intercross line in combination with congenic strains, Jagodic et al. [10] fine mapped *eae18 *and identified two adjacent QTLs, *eae18a *and *eae18b*, on the rat chromosome 10 in a myelin-oligodendrocyte glycoprotein (MOG)-induced, chronic relapsing EAE. The *eae18b *locus is also orthologous to human chromosome 17q11 and encodes a cluster of β-chemokine genes.

The recognition of β-chemokine genes as susceptibility and quantitative trait loci in mouse and rat EAE along with the human data revealing the β-chemokine gene cluster as a susceptibility locus in MS, strongly suggest the involvement of β-chemokine variants in the development of inflammatory demyelination.

## CCL and CCR molecules in inflammatory demyelination

### Experimental allergic encephalomyelitis

EAE is a valuable model for studying the effector arm of immune response in inflammatory demyelination. It can be induced in susceptible strains of inbred and outbred species by active immunization with myelin related proteins and their peptides (myelin basic protein – MBP, proteolipid lipoprotein – PLP, myelin oligodendrocyte glycoprotein – MOG) emulsified in Freund's complete adjuvant along with intravenous Pertussis toxin, or with a passive transfer of myelin antigen specific T cell lines into naïve recipients. Using various immunization protocols, acute and chronic relapsing (CR-EAE) models have been developed. In both the active immunization and the passive transfer models of EAE, the efferent arm of immune response involves the migration of monocytes / macrophages, dendritic cells and activated myelin-antigen-specific T lymphocytes from the blood circulation into the CNS, where a reactivation of specific lymphocytes by myelin-antigen-presenting dendritic cells, macrophages and residential microglia takes place, and the sequential development of perivascular and parenchymal inflammation is followed by demyelination and neuronal degeneration.

Encephalitogenic T lymphocytes have CD4+ TH1 phenotype characterized by the production of interleukin (IL)-2 and interferon-γ. TH2 lymphocytes producing IL4, IL5, IL6 and IL10 cytokines are involved in the counter-regulation of TH1 effects, and promote clinical recovery. The TH1 / TH2 polarization is regulated by cytokines and chemokines. The transmigration of immune competent cells via the blood-brain barrier is aided by a temporal and spatial regulation of adhesion molecules on T lymphocytes and their counterparts on endothelial cells, and of chemokine ligands and their receptors in the residential CNS and hematogenous mononuclear cells.

One of the most comprehensively studied CC chemokines in inflammatory demyelination is MCP-1 (CCL2). MCP-1 (CCL2) influences both innate immunity through its chemoattractant effect on monocytes / macrophages, and adaptive immunity through its effect on T cell polarization towards the TH2 subtype [11]. CCL2 primarily acts via the CCR2 receptor [1].

MIP proteins have both chemotactic and proinflammatory effects, but also promote homeostasis [6]. The MIP-1 family includes MIP-1α (CCL3), MIP-1β (CCL4), MIP-1δ (CCL9/10) and MIP-1γ (CCL15) that are produced by macrophages, microglia, astrocytes, dendritic cells and lymphocytes. These MIP-1 molecules act via CCR1, CCR3 and CCR5 expressed by lymphocytes and monocytes. MIP-1 proteins also regulate immune response by modulating T cell differentiation. The CCL3 and CCR5 interaction promotes polarization towards the TH1 subtype.

Our understanding concerning the role of these CC chemokines and their receptors in inflammatory demyelination was greatly advanced by studies in the EAE model. In mice, the increased expression of MCP-1 (CCL2) by CNS immune cells is closely associated with the clinical activity of EAE [12-14]. Some studies, however, suggest that the presence of leukocytes is necessary for the production of CCL2 by astrocytes, as the expression of CCL2 prior to the accumulation of inflammatory mononuclear cells has not been observed in the CNS. Substantial MCP-1 (CCL2) expression may only occur in the late phase of acute disease and in the relapsing phases of CR-EAE. It was therefore postulated, that CCL2 is involved in the amplification rather than in the initiation of EAE [4]. In contrast, the MIP-1α (CCL3) expression correlates with the severity of acute disease and also is elevated during relapses in CR-EAE. RANTES (CCL5) is expressed in the CNS throughout the course, but does not correlate with the severity of acute or CR-EAE [13].

Jee et al [15] compared the histological features and MCP-1 (CCL2) and CCR2 expression levels in the lesions of Lewis rats during the acute attack of monophasic EAE and during the first two clinical events of CR-EAE. In concert with the mouse data [4,13], not only higher numbers of macrophages infiltrated the spinal cord during the first and second attacks of CR-EAE as compared to those at the peak of acute EAE in these rats, but the expression of MCP-1 (CCL2) was also significantly higher in the lesion of CR-EAE as compared to that of acute EAE. Similarly, CCR2, the main receptor for CCL2, was expressed by astrocytes, macrophages and T cells in higher amounts during CR-EAE than at the peak of acute EAE. This observation confirmed the role of CCL2 – CCR2 interaction in the development of relapses.

Youseff et al [16] observed an increased mRNA transcription not only for MCP-1 (CCL2), but also for MIP-1α (CCL3) and MIP-1β (CCL4) at the onset of EAE in rat brains. MIP-1α (CCL3) and MCP-1 (CCL2) declined in two days even though the clinical disease further progressed. MIP-1β (CCL4) mRNA declined in correlation with the clinical recovery. RANTES (CCL5) mRNA, in contrast, increased in the brains only after recovery. The full length, reverse transcribed and PCR amplified DNA product for each of these four CCL molecules was transferred into a plasmid vector and injected as naked DNA vaccine into rats. Both the transcription of a relevant chemokine and the induced antibody response against it was monitored. The *in vivo *immune response to these CCL molecules differentially influenced the evolution of EAE. MIP-1α (CCL3) and MCP-1 (CCL2) DNA vaccines prevented EAE, while MIP-1β (CCL4) aggravated the disease and RANTES (CCL5) did not have an effect on the course of EAE. This study emphasizes the importance of CCL2 and CCL3 in the development of active EAE in rats.

CCL1 also attracted attention in EAE. Teutscher et al [9] identified *eae7 *encoding CCL1 and other chemokines as a susceptibility locus and QTL in murine EAE. SNPs in CCL1 differentially segregated in mouse strains susceptible or resistant to EAE. mRNA molecules for both CCL1 and its receptor CCR8 were detected in spinal cord lesions of EAE, in correlation with the expression of tumor necrosis factor (TNF)-α by inflammatory leukocytes [17-19]. As both CCL1 and CCR8 were detected in microglia, an autocrine signaling mechanism was postulated. CCR8 (-/-) mice showed marked delay in the onset and reduced severity of EAE as compared to controls. Leukocyte infiltration in the spinal cord was not diminished in the CCR8 (-/-) mice, suggesting that that a defective microglial activation might have altered the clinical phenotype.

Recent studies addressed the role of chemokines at the blood-brain barrier. Using intravital fluorescence videomicroscopy, Vajkoczy et al [20] demonstrated that the interaction between encephalitogenic T cells and endothelial cells of the BBB involves α4-integrin (VLA-4) which mediates a G-protein-independent capture (arrest) followed by G-protein-dependent adhesion strengthening of circulating T cells to VCAM-1 on endothelial cells. Postulating the involvement of chemokines in the integrin-mediated arrest of autoreactive T cells at the BBB, the investigators [3] subsequently aimed to identify the specific chemokines by performing *in situ *hybridization and immunohistochemistry on brain and spinal cord sections of mice with EAE. Constitutive expression of the lymphoid chemokine called EBV-induced molecule 1 ligand chemokine (ELC) / CCL19 in a subpopulation of CNS venules and induced expression of the secondary lymphoid chemokine (SLC) / CCL21 in inflamed CNS venules was detected. CCR7, the common receptor for these two chemokines was expressed on a subpopulation of cells in the perivascular cuffs. Encephalitogenic T cells *in vitro *showed expression of CCR7 and CXCR3, the alternative receptor for CCL21, and chemotaxed towards both CCL19 and CCL21 in a concentration-dependent and a Pertussis toxin-sensitive manner similar to naïve T cells. Functional deletion of CCR7 and CXCR3 or immune blockade of CCL19 and CCL21 reduced the binding of encephalitogenic T cells to inflamed venules in frozen brain sections. Altogether, these data suggest that CCL19 and CCL21 are expressed in cerebral endothelial cells and are involved in α4-integrin mediated adhesion strengthening of autoreactive T cells and subsequently of other inflammatory cells to the endothelial layer of the BBB. These molecular interactions may lead to permanent inflammatory cell immigration into the CNS in chronic autoimmune disease.

CCL20 or MIP-3α (exodus-3 / LARC) is a chemokine active on dendritic cells and lymphocytes that express CCR6 [1]. Serafini et al [21]demonstrated the occurrence of dendritic cells in the spinal cord of mice immunized with the PLP139–151 peptide. Although dendritic cells were present during early acute, chronic and relapsing EAE, most prominent infiltration of spinal cord by mature dendritic cells was noted in relapsing disease. In all stages of EAE, CCL20 and CCR6 were upregulated in the CNS. This study emphasizes the importance of dendritic cells in antigen presentation and T cell restimulation, and links the immigration of dendritic cells to the expression of CCL20 in the CNS during EAE.

CCL22 or macrophage-derived chemokine (MDC) is chemoattractant for monocytes, dendritic and NK cells, and T lymphocytes of the TH2 subtype. MDC / CCL22 acts via CCR4 which is preferentially detected on TH2 type, memory and regulatory T cells [22]. While MDC / CCL22 is considered to be predominantly involved in TH2 mediated immunity, Columba-Cabezas et al [22] demonstrated mRNA expression for MDC / CCL22 in the CNS of mice with relapsing-remitting and chronic-relapsing EAE induced by PLP139–151 or whole spinal cord homogenate. Immunohistochemistry demonstrated that MDC / CCL22 is produced by infiltrating leukocytes and residential microglia, while CCR4 is expressed by infiltrating leukocytes. *In vitro *activation of microglia resulted in secretion of bioactive MDC / CCL22 that induced chemotaxis of TH2 lymphocytes. This study concludes that MDC / CCL22 produced by microglia may play a role in a TH1 mediated CNS inflammation by inducing the homing of TH2 regulatory cells into the lesion site.

To further clarify the role of chemokine receptors involved in EAE, Fife et al [23] examined CCR expression in normal (unprimed), PLP139–151 primed non-activated, PLP139–151 primed and reactivated lymph node derived T cells, and CNS-isolated CD4+ T cells from SJL mice receiving PLP139–151 specific, *in vitro *reactivated T cells. Normal resting CD4+ T cells and primed non-activated T cells expressed mRNA for CCR1, CCR2, CCR3, CCR5, CCR6, CCR7 and CCR8. *In vitro *activated T cells expressed in higher amounts most of the CCRs found in normal T cells as well as CCR4. After passive transfer of encephalitogenic activated T cells in naïve recipients, the donor derived encephalitogenic cells and the host-derived CD4+ T cells isolated only from the CNS lesions but not from spleen expressed mRNA for CCR1. This latter observation was confirmed at protein level, and appeared to be specific for acute EAE. Neutralization of the CCR1 ligand CCL3 (MIP-1α) diminished the inflammatory infiltrate in the CNS.

The effects of anti-chemokine treatments in the mouse EAE is summarized by Elhofy et al [24] and Karpus et al [25] and is in consensus with data in the rat model. Although various strains and protocols were used, overall anti-RANTES (CCL5) had no effect in these models, anti-MIP-1α (CCL3) decreased the severity of acute EAE and anti-MCP-1 (CCL2) reduced the severity of both acute EAE and the relapses in CR-EAE. However, it is important noting that in some respect, these observations are model specific. While the impact of anti-CCL5 immune treatment was unremarkable in the autoantigen-induced forms of EAE, antibody treatment targeting CCL5 in a mouse hepatitis virus-induced inflammatory demyelination model resulted in diminished leukocyte infiltration and reduced neurological disability [26].

Genetic manipulations of the murine model provide further insights in the characterization of CCL / CCR molecules in EAE. In mice with the CCL2 transgene under the control of the *lck *(which directs the expression of transgene to cortical thymocytes) or MBP promoters (which directs the expression of transgene to the CNS), a spontaneous infiltration of monocytes / macrophages in the thymus and CNS was observed, respectively [27]. LPS injection induced higher CCL2 expression in the brain and markedly enhanced the mononuclear cell (MNC) infiltrate. The relationship between LPS treatment, CCL2 expression and MNC recruitment into the CNS remains partially understood, and seems to involve a complex immune regulatory mechanism rather than just a selective effect mediated by the upregulation of the CCL2 transgene. Nevertheless, these transgenic mice were clinically normal both before and after LPS injection. More recently, Elhofy et al [28] examined TH1 lymphocytes in a PLP-induced EAE model using a transgenic mouse strain that constitutively expressed low CCL2 levels in the CNS under the control of the astrocyte-specific glial fibrillary acidic protein promoter. CCL2 transgenic mice developed milder EAE than the littermate controls, despite similar numbers of CD4 and CD8 T cells in the CNS infiltrates and an increased number of monocytes in the CNS of the CCL2 transgenic animals. Functional studies revealed that encephalitogenic T cells from the CCL2 transgenic mice produced significantly less interferon-γ and proliferated less in the presence of PLP peptides than those of the non-transgenic controls. Increased CCL2 expression in the CNS also resulted in a decreased IL-12 receptor expression by PLP-specific T cells. Thus in this model, the overexpression of CCL2 in the CNS resulted in a suppression of the TH1 response and a milder clinical phenotype of EAE, despite the enhanced effect on monocytes.

The CCL2 knock out (-/-) mice showed resistance to EAE and significantly decreased macrophage infiltration in the CNS following active immunization with MOG35–55 peptide. While T cells from CCL2 (-/-) mice transferred EAE to wild type mice, wild type T cells did not induce EAE in CCL2 (-/-) recipient mice. These observations suggest a key role for CCL2 in the recruitment of macrophages into the CNS and thus, in the pathogenesis of EAE [29,30,4]. The array of ligands for CCR2 includes MCP-1 (CCL2), MCP-2 (CCL8), MCP-3 (CCL7) or MCP-5 (CCL12). As CCL2 (-/-) mice did not show a compensatory upregulation of MCP-2 (CCL8), MCP-3 (CCL7) or MCP-5 (CCL12) mRNA molecules, MCP-1 (CCL2) is likely to be the main ligand for CCR2 in mice with EAE.

The clinical phenotype of CCR2 (-/-) genotype was similar to that of the CCL2 (-/-) genotype, characterized by a reduced macrophage infiltration in the spinal cord and a decreased susceptibility to actively (MOG35–55) induced acute EAE in the studies by Fife et al [31] and Izikson et al [32]. T cells from CCR2 (-/-) immunized mice produced similar levels of interferon-γ and IL2 as those from controls, and were capable of transferring EAE in a naïve recipient. In contrast, T cells from wild type mice did not cause EAE in a CCR2 (-/-) recipient [31]. However, these observations again appeared to be model specific. Gaupp et al [33] reported that, even though the disease was milder or delayed, three CCR2 (-/-) mouse strains retained susceptibility to EAE in their experiments. Histological analyses revealed an abundance of neutrophils in lesions of the CCR2 (-/-) mice in contrast to the monocyte abundance in EAE lesions of wild-type mice. The development of compensatory immune mechanisms for the lack of CCR2 was evidenced by the increased mRNA expression for other CCL and CCR molecules (most notably IL8 and its receptor involved in neutrophil recruitment). This study emphasizes that promiscuity of chemokines and their receptors may overcome the deletion of a single CCR receptor with a resultant mild modification of the clinical and more profound modification of the histological phenotype.

Further studies demonstrated an approximately 50% reduction of clinical EAE activity in the CCR1 (-/-) mice, likely involving the altered migration of monocytes and lymphocytes [34]. In contrast to the observed EAE suppression in the CCR1 (-/-) and CCR2 (-/-) models, the CCR5 knockout mice had the same disease severity as the wild-type controls [35]. These studies underscore the importance of CCR1 and CCR2 in the development of inflammatory demyelination and give support to novel alternative strategies targeting these CCR molecules. Such strategies include the development of small functional CCR antagonists, amongst which the most significant progress has been made with CCR1 antagonists [36,37]. CCR1 antagonist compounds were shown to inhibit CCL3 and CCL5 induced migration of MNCs in a dose dependent manner, and to reduce clinical EAE in rat [36-38].

In sum, CCL and CCR data from rodent EAE models using inbred, transgenic and knockout strains along with data from chemokine-specific antibody treatments or CCL DNA immunization in EAE suggest that concentration gradients of CCL2 and CCL3 decreasing from the CNS to the peripheral circulation are involved in the spatially and temporally regulated recruitment of mononuclear cells into the CNS which correlates with the course of clinical disease. CCL2 may play a more significant role during relapses than during the induction phase of the disease. CCL5 is expressed by mononuclear cells in the perivascular space during the recovery phase of an acute event, and may therefore be involved in the regulation of recovery rather than in the initiation of the disease. In addition, CCL19 and CCL21 are expressed by endothelial cells of the BBB, and are involved in the strengthening of leukocyte adhesion to inflamed venules followed by homing of encephalitogenic T lymphocytes to the CNS. CCL20 can control the recruitment of dendritic cells into lesions, whereas CCL22 may be involved in a TH2 mediated regulatory process during EAE. CCL1 is likely playing an important role in the autocrine regulation of activation of macrophages and microglia in EAE lesions. Thus, the functional involvement of CCL chemokines during EAE is not only restricted to a well orchestrated recruitment of dendritic cells, monocytes, macrophages, T effector and regulatory cells into the CNS, but also includes a temporal and spatial regulation of TH1 (CCL3, CCL5) or TH2 (CCL2, CCL22) polarization, and monocyte, macrophage and microglial activation (CCL1, CCL2, CCL7, CCL8). Their receptors, the CCRs play equally important roles in these processes. Experimental evidence now suggests that CCR1, CCR2, CCR3, CCR4, CCR5, CCR6, CCR7, CCR8 and CXCR3 on hematogenous mononuclear cells recognize these chemoattractant and regulatory molecules to induce cell differentiation, adhesion or migration of distinct inflammatory cells in peripheral lymphoid organs, at the BBB and in the CNS during the course of EAE. Even taking into consideration the complex and promiscuous nature of the CCL – CCR network, certain pathways may be associated with distinct biological function amenable to intervention. Targeting CCR molecules either by monoclonal antibodies or by small functional antagonists has become a novel and realistic strategy in the treatment and prevention of autoimmune diseases.

### Multiple sclerosis

The complexity of disease pathogenesis, difficulties accessing the site of pathology and the descriptive nature of studies explain why the available CCL / CCR data are less comprehensive in MS as compared to those in EAE. Nevertheless, new observations support the generally accepted views that MS is a predominantly TH1 lymphocyte mediated disease, and CCL – CCR molecules play a significant part in the regulation of intercellular interactions in the peripheral lymphoid organs, at the BBB and in the CNS. In addition to defining chemotaxis, CCL-CCR interactions are involved in TH1 / TH2 polarization and regulation in MS. Recent studies also raise the possibility that distinct molecular mechanisms with characteristic CCL-CCR kinetics correlate with the development of histological subtypes of the disease.

#### CCRs in the multiple sclerosis brain

A recent review of chemokines and their receptors [39] suggests that every CC chemokine receptor (CCR1-CCR5) interact with multiple CCLs and vice versa. Five CCRs (CCR1, CCR2, CCR3, CCR5 and CXCR3) were detected on infiltrating monocytes, macrophages and lymphocytes in MS lesions. In contrast, several members of the CCL family [CCL2 = MCP-1, CCL3 = MIP-1α, CCL4 = MIP-1β, CCL5 = RANTES, CCL7 = MCP-3, CCL8 = MCP-2] were expressed in astrocytes, microglia and other inflammatory cells within MS lesions.

While control brain specimens had only scarce appearance of CCR positive (microglial) cells throughout the CNS, foamy macrophages, microglia, perivascular lymphocytes and occasionally, astrocytes were positive for CCR2, CCR3 and CCR5 in chronic active plaques [39,40]. In other studies, CCR1, CCR2, CCR3 and CCR5 were detected on mononuclear cells and macrophages in demyelinating plaques [41,42].

Trebst et al [43] investigated the kinetics of CCR expression. In early demyelinating lesions, CCR1+/CCR5+ hematogenous monocytes and CCR1-/CCR5- microglial cells were detected. In later stages, macrophages became CCR1-/CCR5+, while microglia upregulated CCR5. This observation suggest that CCR1+/CCR5+ hematogenous monocytes enter into the CNS and stay there in the presence of appropriate ligands. During evolution of lesions, these cells down-regulate CCR1 while retain the CCR5 expression. A more recent study [44] reveals that this distinct temporal pattern, namely the decrease in CCR1+ and increase in CCR5+ cells, may be restricted to the histological type II demyelinating lesions characterized by mononuclear cell infiltration and immunoglobulin plus complement deposition, and is not seen in type III lesions characterized by oligodendrocytopathy and apoptosis [45].

CCR2 may also play a key role in the lesion development based on more indirect information. CCR2 is the main, but not exclusive, functional receptor for CCL2 [4], and as discussed above, the CCL2 – CCR2 interaction appears to play a key role in the development of EAE lesions. Microglia, macrophages and perivascular mononuclear cells show some degrees of immune reactivity for CCR2 in chronic active plaques in several studies, but the expression of CCR2 is generally low in MS lesions. Nevertheless, the data from EAE and observations in MS suggest that the CCR2 – CCL2 interaction is important in the development of plaques. This view was recently proposed and will be discussed below.

CCR8, the receptor for CCL1, has been detected *in vitro *on TH2 and regulatory lymphocytes, macrophages and microglia. Using immunohistochemistry, Trebst et al [19] detected CCR8 on phagocytic macrophages and activated microglia in type II and type III demyelinating MS lesions. CCR8 expression correlated with the demyelinating activity, but was not restricted to the MS pathology. Phagocytic macrophages and activated microglia in stroke and progressive multifocal leukoencephalopathy also expressed CCR8. Thus, CCR8 seems to identify a subset of activated microglia in different CNS pathologies.

#### CCLs in the multiple sclerosis brain

Using methods of immunohistochemistry and *in situ *hybridization, McManus et al [46] investigated the expression of three monocyte chemoattractant proteins, MCP-1 (CCL2), MCP-2 (CCL8) and MCP-3 (CCL7) in correlation with the temporal evolution of plaques. All three proteins were detected in high amounts in the center, but sharply decreased at the edges of acute and chronic active lesions. Hypertrophic astrocytes showed the strongest expresion, while infiltrating mononuclear cells showed variable reactivity in plaques. MCP-3 (CCL7) was also detected in the extracellular matrix. Reactivity for these CC chemokine ligands outside of plaques was otherwise restricted to hypertrophic astrocytes. *In situ *hybridization confirmed the observation for CCL2 at mRNA level. There seemed to be an inverse correlation between the age of plaques and expression of these three CCL molecules, with only a scanty appearance of immunoreactive astrocytes in chronic silent lesions. These methods did not detect MCP chemokines in the brains of normal controls.

Additional studies demonstrated the expression of CCL3 and CCL4 in macrophages and microglia, and CCL3 also in astrocytes [47-49]. CCL5 was primarily detected in perivascular inflammatory cells and astrocytes [48-50].

While most of the above studies used the method of immunohistochemistry, we recently assessed the mRNA expression levels for CCL2, CCL3, CCL5, CCL7, CCL8, CCL13 and CCL15 relative to β-actin in corresponding normal appearing white matter (NAWM), normal appearing gray matter (NAGM) and chronic active plaque containing specimens from ten post mortem MS brains. These specimens were characterized by hematoxyllin & eosin, Luxol Fast Blue and immune staining specific for CD68 and β_2_-microglobulin [51]. In addition, the expression distribution for pro- and anti-apoptotic molecules in these specimens was also assessed by real-time PCR [51]. The selection of the above listed CC chemokines was based on two considerations. First, we detected MS associated SNP haplotypes in the genes of CCL2, CCL11-CCL8-CCL13, CCL15 and CCL3 [8]. Second, previous studies suggested the involvement of CCL2, CCL7, CCL8, CCL5 and CCL3 molecules in the development of plaques [39,46]. While neither our genetic nor our mRNA studies revealed positive findings for CCL5, the three MCP chemokines CCL2 (MCP-1), CCL7 (MCP-3) and CCL8 (MCP-2) showed altered regional expressions in MS brains. We detected an increased expression of CCL2 in plaques as compared to NAWMs, and an increased expression of CCL7 in both plaques and NAWMs as compared to NAGMs. In contrast, the expression level of CCL8 was decreased in plaques as compared to NAWM or NAGM specimens (Banisor and Kalman, unpublished observation). This analysis of CCL mRNA molecules in various regions of MS brain complements the data from previous immunohistochemical studies, and further confirms the involvement of CCL2 and CCL7 (and possibly of CCL8) in the development of pathology. In consensus with others, however, we also note an increased CCL7, CCL8 and CCL13 expression in the white matter as compared to the gray matter in 5 other neurological disease controls (1 viral and 1 post-infectious encephalitis, 2 Alzheimer disease and 1 Parkinson disease). No differences were observed for any of these molecules in the white and gray matters of normal controls. We postulate that the expression of CCL molecules may be detected in various inflammatory conditions of the CNS, however, the temporal and cell specific upregulation of certain CCL and CCR molecules is pathology specific. Therefore, further exploration of the expression kinetics of these molecules may facilitate a better understanding of MS pathogenesis.

#### CCL and CCR detected in blood and CSF

Relatively limited numbers of studies are available regarding chemokines and their receptors in the blood circulation and in the cerebrospinal fluid (CSF) in MS patients. The expression of CCR5 was found to be higher on circulating T lymphocytes from MS patients than on those from normal controls. These T cells showed an increased migration towards CCL3 and CCL5, suggesting a functional significance of the altered receptor expression [42,52]. The migratory population represented predominantly TH1 / TH0 cells, while the non-migratory population was enriched for TH2 cells. The aberrant migration of T cells towards CCL3 and CCL5 was related to the increased expression of the CCR5 receptor, and could be blocked by anti-CCR5 antibodies. A fluctuation of CCR5 expression by T cells was also suggested in correlation with relapses and remissions in a small group of patients [53].

Sorensen and Sellebjerg [54] assessed the CCR expression profile on peripheral T cells of patients with relapse, remission or secondary progressive disease, and detected a higher percentage of CCR2-expressing T cells in secondary progressive MS (SPMS) than in other patient groups. CCR2-positive T cells displayed TH2 profile producing IL5 and tumor necrosis factor-α. The CCR5 expression associated with TH1 profile was significantly lower in SPMS than in patients with relapsing-remitting MS (RRMS) during relapse. Thus, the authors conclude that patients with SPMS have a high expression of CCR2, a chemokine receptor associated with TH2 profile, whereas patients with RRMS preferentially display T cells with CCR5 expression and TH1 profile. More CCR5 positive T cells produced tumor necrosis factor-α in patients with RRMS than those in patients with SPMS. CCR2 is known to be predominantly expressed on monocytes. However, when expressed on T cells, CCR2 is associated with the TH2 subtype as CCL2 induces differentiation of T cells into TH2 phenotype [11]. While Sorensen and Sellebjerg [54] detected significant differences in the CCR5 and CCR2 expression profile between RRMS and SPMS, they noted no differences in CCR expression between RRMS and controls. The observation regarding the association of CCR5 with RRMS is consistent with a previous study revealing that patients with the defective CCR5 receptor (CCR5 Δ32 deletion) have prolonged relapse free periods, but the long term prognosis of MS did not seem to correlate with the CCR5 Δ32 genotype [55]. Besides establishing the CCR characteristics in RRMS and SPMS, this study also suggests that targeting the CCL2-CCR2 axis with specific CCR2 antagonist or a combination of CCR2 and CCR5 antagonists might be an option in SPMS, whereas CCR5 antagonists alone may be considered in RRMS.

However, CCR5 on peripheral MNCs was not uniformly found to be differentially expressed in MS subtypes [56]. MNCs from blood constitutively expressed CCL4 and CCL5, the ligands for CCR5, in all patient groups and controls. This study also failed to detect CCL2 and CCL3 by ribonuclease protection assay in peripheral blood MNCs. Further, the complexity of information regarding CCR5 is reflected by a recent study suggesting the association of the CCR5 Δ32 genotype with early death in MS [57].

There is relatively limited information available regarding CCR and CCL expression levels in the CSF. Some investigators showed that CCR5+ mononuclear cells of MS patients were enriched in the CSF, representing a significant proportion of monocytes and only a minority of T cells. However, neither cell population differed quantitatively from those of controls, suggesting that CSF leukocytes may not be fully reflective of CNS inflammation [39,55].

Giunti et al [58] detected CCR5, CCR7 and CXCR3 positive T cells in the CSF of patients with MS and other inflammatory neurological disease (IND) (meningitis, encephalitis, CIDP, neuroborreliosis). Coexpression of these receptors was noted on a subset of memory cells. The increased ratio of CXCR3 / CCR4 was suggested as a molecular correlate of disease activity by Nakajima et al.[59] TH1 clones established from the CSF of patients with IND and of controls similarly migrated *in vitro *towards CXCL10, CXCL12 and CCL5. CXCL10, CXCL12 and CCL19 were increased in the CSF of these patients [58].

Amongst CC chemokines, CCL3 and CCL5 were most consistently found to be elevated in the CSF of MS patients during relapses as compared to normal controls [59-61]. In contrast, decreased CCL2 was found in the CSF in all clinical forms of MS by Scarpini et al.[62] More consistently, however, low CCL2 levels were detected only during relapses by others [41,59,61,63,64]. The drop of CCL2 in the CSF was not found during relapses of neuromyelitis optica [65]. Mahad et al [64] also found that CCL2 in the CSF was decreased not only in patients with MS but also in patients with IND when compared to those of non-inflammatory CNS disease controls. In contrast, Bartosik-Psujek and Stelmasiak [61] observed an increase in both CCL2 and CCL5 in the CSF of patients with IND, and suggested that the drop of CCL2 during relapses is characteristic only of MS. Further, CCL2 concentration increased as time from the last relapse increased and following corticosteroid therapy [63,64]. With the exception of well defined changes in the CCL2, CCL3 and CCL5 levels in the CSF during relapses, most investigators observed no differences in various clinical forms of the disease [56,61,64,66].

Pashenkov et al [67] studied two secondary lymphoid organ chemokines, CCL19 (exodus-3, MIP-3β) and CCL21 (exodus-2, SCL) in CSF and sera of patients with MS, clinically isolated syndrome (CIS) presenting as optic neuritis (ON), isolated ON, IND and non-inflammatory neurological disease controls (NINC). CSF of the NINC group contained CCL19 but not CCL21, while both chemokines were elevated in the CSF of patients with MS, CIS-ON and IND. The authors postulate that CCL19 and CCL21 may control the retention of dendritic cells and the recruitment of naïve T cells and activated B cells, or a de novo formation of lymphoid structures in plaques. These cells are known to express CCR7, the receptor for CCL19 and CCL21. EAE studies also support the notion that CCL19 and CCL21 play important roles both at the BBB and in the CNS [3].

To correlate previous data on CCL concentrations in the CSF of MS patients, Kivisakk et al [68] measured mRNA for CCL2 / MCP-1 and CCL5 / RANTES in MNCs in the CSF and blood of patients with MS, acute meningitis and normal controls. While high numbers of MNCs expressing CCL2 and CCL5 were found in some patients, overall no differences were observed between MS and acute meningitis. This study would argue that there is no systemic dysregulation of CC chemokines contributing to MS pathogenesis.

In sum, the above data suggest that CCL1, CCL2, CCL3, CCL4, CCL5, CCL7 and CCL8 are expressed by residential glia and perivascular leukocytes in plaques. Expression of the corresponding CCR1, CCR2, CCR3, CCR5 and CCR8 receptors has been demonstrated on infiltrating leukocytes, but also on microglia, dendritic cells and astrocytes. While the expression kinetics of CCR1 and CCR5 may discriminate between histological type II and type III lesions of MS, CCR8 is similarly expressed in both lesions types (Table 1).

**Table 1:** CCR and CCL molecules in plaques, blood and CSF of MS patients. This Table summarizes in a cross-sectional manner major findings regarding CCR and CCL expression in brain, blood and CSF of multiple sclerosis patients. The dynamic nature of changes is detailed in the text. The interactions of CCL-CCR molecules on specific cell types are depicted in Figure 1. Type II and III lesions refer to the histological classification proposed by Lucchinetti et al [45]. References are indicated in brackets.

	**In chronic active plaque expressed on**	**In blood expressed on**	**In CSF expressed on**
CCR1	Monocyte, macrophage, lymphocyte [39,41,42]		
CCR2	Monocyte, macrophage, lymphocyte [39-42]	TH2 in SPMS [54]	
CCR3	Monocyte, macrophage, lymphocyte [39-42]		
CCR4	Monocyte, macrophage, lymphocyte [39]		
CCR5	Monocyte, macrophage, lymphocyte [39-42]	TH1/TH0 in RRMS [42, 52-54]	MNC [39,55,58]
CCR7			T, dendritic [58]
CCR8	Macrophage, microglia in type II and III lesions [19]		

	**In early -> late stage type II lesion**		

CCR1+/CCR5+ -> CCR1-/CCR5+ Monocyte, macrophage [43]			
CCR1-/CCR5- -> CCR1-/CCR5+ Microglia [43]			

	**In acute, and to lesser degrees, in chronic active plaques expressed by**	**In blood expressed by**	**In CSF**

CCL2	Astrocyte, microglia, MNC [46]		low in relapse [59,61,63,64]
CCL3	Astrocyte, microglia, macrophage [47-49]		increased in relapse [59-61]
CCL4	Microglia, macrophage [47-49]	MNC [56]	
CCL5	MNC, astrocyte [48-50]	MNC [56]	increased in relapse [59-61]
CCL7	Astrocyte, microglia, MNC [46]		
CCL8	Astrocyte, microglia, MNC [46]		
CCL19			present in NIND, increased in MS, CIS-ON, IND [67]
CCL21			increased in MS, CIS-ON, IND [67]

The increase of CCL3 and CCL5 in the CSF during a relapse correlates with the increase in the expression of their receptor, CCR5 on TH1 lymphocytes, which results in an enhanced migratory activity of these cells towards CCL3 and CCL5. The consistently observed decrease in CCL2 levels in the CSF during or even prior to a relapse generated alternative considerations. The first consideration suggests, that the decreased CCL2 level likely relate to a decreased TH2 lymphocyte activity, as CCL2 induces TH2 polarization. Vice versa, CCL2 expression is controlled by TH2 cytokines such as IL4. The concept of CCL2 – TH2 coregulation is supported by the observation that clinical improvement and normalization of the inflammatory CSF profile after corticosteroid treatment correlate with the normalization of CCL2 in the CSF. Thus, measurements of CCL2 in the CSF may also reflect the fluctuation of TH2 activity during the course of MS. The second consideration was proposed by Dr. Ransohoff (oral presentation at the ECTRIMS meeting 2004) [4,69]. This interpretation reconciles the complex observations from the EAE model suggesting a key role for CCR2 – CCL2 in the development of inflammatory lesions, and from MS suggesting a low expression of CCR2 and increased expression of CCL2 in active plaques, but a decreased CCL2 level in the CSF. Based on this model: 1) CCL2 – CCR2 play an important role in the development of inflammatory demyelinating lesions both in EAE and MS; 2) CCL2 expressed in the CNS attracts CCR2+ monocytes and T cells into the developing plaque; 3) while CCR2 binds and internalizes CCL2 molecules in large amounts, CCL2 will be consumed resulting in a reduced CCL2 level in the intercellular fluids and the CSF; 4) when CCR2 encounters its ligand, the CCR2 / CCL2 complex will be internalized and CCR2 will be downregulated on the surface of inflammatory cells in the lesion.

## Conclusion

**Figure 1:**
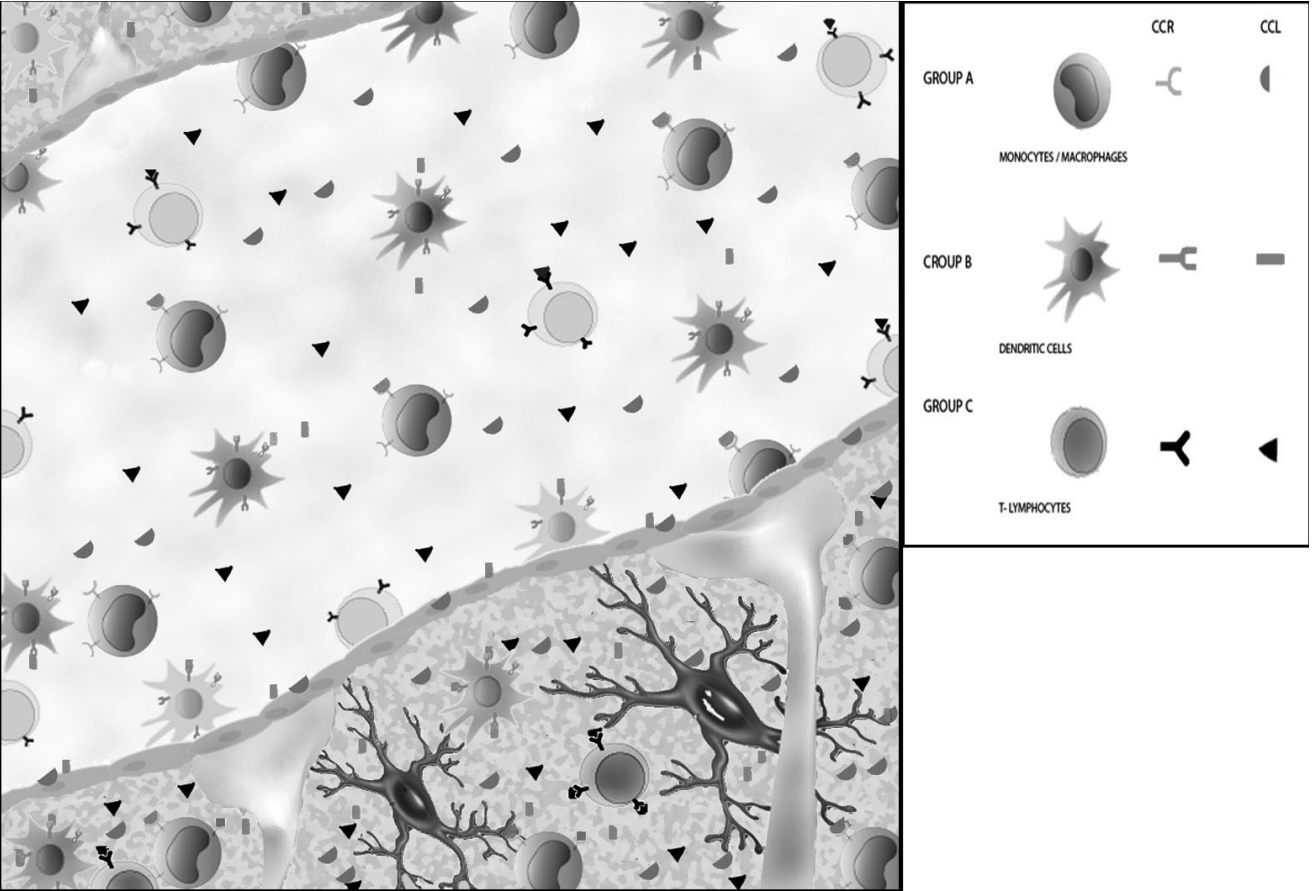
**Interaction between CCL and CCR molecules at the blood-brain barrier.** This figure depicts CCL-CCR interactions at the BBB (endothelial cells and astrocytic processes) interfacing a venule and the CNS. CCL molecules (most prominently CCL2, CCL3, CCL7 and CCL8, but also CCL1, CCL4, CCL19 and CCL21) are produced by residential microglia, astrocytes and endothelial cells throughout the course of lesion development, and by infiltrating MNCs (CCL5) during late phases of plaque formation, and attract functionally different subsets of monocytes / macrophages, dendritic cells and T lymphocytes from the circulation via the BBB into the CNS. The temporal and spatial regulation of molecular events, the association of distinct CCR molecules with different histological subtypes of demyelination and the involvement of different CCL-CCR interactions in T cell polarization are detailed in the text. Here we illustrate in a simplified and cross-sectional manner the main groups of interacting receptors on various hematogenous cells and ligands released by residential immune cells of the CNS or by components of the BBB. Group A of receptors and ligands expressed by and acting on monocytes / macrophages, respectively: CCR1 / CCR2 / CCR3-CCL7, CCR2-CCL2, CCR3-CCL8, CCR4-CCL22; Group B of receptors and ligands expressed by and acting on dendritic cells, respectively: CCR4-CCL22, CCR6-CCL20, CCR7-CCL19 / CCL21; Group C of receptors and ligands expressed by and acting on T lymphocyes, respectively: CCR1-CCL3 / CCL5, CCR2-CCL2, CCR4-CCL22, CCR5-CCL3 / CCL4 / CCL5, CCR7-CCL19 / CCL21, CCR8-CCL1.

Studies on EAE and MS suggest that CC chemokine ligands (most prominently CCL2, CCL3, CCL5, CCL7, CCL8, but also CCL1, CCL4, CCL19 and CCL21) expressed by residential immune cells in the CNS or by endothelial cells at the BBB are major chemoattractants for hematogenic immune cells (primarily monocytes / macrophages [CCL2, CCL7, CCL8, CCL22] dendritic cells [CCL19, CCL20, CCL21, CCL22] and T lymphocytes [CCL1, CCL2, CCL3, CCL4, CCL5, CCL19, CCL21, CCL22]) via interactions with their G-protein-coupled receptors (CCR1-CCR10). These CCL – CCR interactions play a key role in the recruitment, activation and retention of immune competent cells in the CNS, with the CCL1 – CCR8, CCL2 – CCR2, CCL3 – CCR1 / CCR5, CCL5 – CCR1 / CCR5, CCL7 – CCR1 / CCR2 / CCR3, CCL8 – CCR3, CCL20 – CCR6, CCL19 / CCL21 – CCR7, CCL22 – CCR4 interactions being the best characterized among them (Figure 1). The EAE model suggests that CCL19 and CCL21 produced by endothelial cells induce G-protein-mediated signaling via their receptor CCR7. This signaling leads to an enhanced adhesion of the leukocyte α4-integrin (VLA-4) to the endothelial VCAM-1 and results in a facilitated transmigration of leukocytes via the BBB. CCL-CCR interactions also define the differentiation and chemotaxis of T cell subpopulations, and thus may control the dynamic changes in the local balance of TH1 (CCL3 – CCR1 / CCR5, CCL5 – CCR1 / CCR5) and TH2 (CCL1 – CCR8, CCL2 – CCR2, CCL22 – CCR4) cell populations in lesion. Different CCL – CCR expression kinetics may characterize the different (initial, height, self-limiting) phases and histological subtypes (type II or type III) of inflammatory demyelination. This differential involvement of chemokines and their receptors in various stages and forms of MS, and the arising information concerning the involvement of genetic variants of CCLs suggest that small CCR antagonists may represent a realistic strategy in controlling the inflammatory activity that may have to be adjusted to individual disease characteristics.

## List of abbreviations

BBB – blood brain barrier

CCL – CC chemokine ligand

CCR – CC chemokine receptor

CIS – clinically isolated syndrome

CNS – central nervous system

CR-EAE – chronic-relapsing EAE

CSF – cerebrospinal fluid

EAE – experimental allergic encephalomyelitis

EBV – Epstein-Barr virus

IL – interleukin

IND – inflammatory neurological disease

LPS – Lipopolysaccharide

MBP – myelin basic protein

MCP – monocyte chemotactic protein

MIP – macrophage inflammatory protein

MNC – mononuclear cells

MOG – myelin-oligodendrocyte glycoprotein

MS – multiple sclerosis

NAGM – normal appearing gray matter

NAWM – normal appearing white matter

NF1 – neurofibromatosis, type I

NINC – non-inflammatory neurological disease controls

NK cells – natural killer cells

NOS2A – nitric oxide synthase 2A

NPL – nonparametric linkage

OMG – oligodendrocyte-myelin glycoprotein

ON – optic neuritis

PCR – polymerase chain reaction

PLP – proteolipid lipoprotein

QTL – quantitative trait loci

RANTES – regulated upon activation normally T expressed and secreted cytokine

RRMS – relapsing-remitting MS

SNP – single nuclear polymorphism

SPMS – secondary-progressive MS

TH1 – T-helper 1

TH2 – T-helper 2

TNF – tumor necrosis factor

VCAM-1 – vascular cell adhesion molecule-1

VLA-4 – very late antigen-4

## Competing interests

The author(s) declare that they have no competing interests.

## Authors' contributions

Ileana Banisor, research assistant, was involved in the acquisition and analyses of our research data mentioned in the paper. She prepared the figure. Thomas P. Leist, collaborator, critically reviewed and edited the manuscript. Bernadette Kalman, P.I., designed the research studies mentioned from her lab, supervised the work processes, interpreted the data and drafted this manuscript. She also generated funding supports.
